# The Many Difficulties and Subtleties in the Cognitive Assessment of Chronic Hepatitis C Infection

**DOI:** 10.1155/2020/9675235

**Published:** 2020-03-19

**Authors:** Jefferson Abrantes, Daniel Simplicio Torres, Carlos Eduardo Brandão – Mello

**Affiliations:** ^1^Department of Neurology, Federal University of the State of Rio de Janeiro, Rua Mariz e Barros, 775 - Tijuca, Rio de Janeiro 20270-004, Brazil; ^2^Department of Gastroenterology, Federal University of the State of Rio de Janeiro, Brazil

## Abstract

Since the discovery of HCV in 1989, several diseases have been related to chronic infection by this virus. Often, patients with hepatitis C virus (HCV) complain of cognitive impairment even before the development of hepatic cirrhosis, which they described as “brain fog.” Several studies have proposed a link between chronic HCV infection and the development of cognitive alterations, but the inclusion of confounding factors in their samples significantly limits the analysis of the results. In this article, we will give an overview about cognitive dysfunction in patients with HCV.

## 1. Introduction

The occurrence of cognitive alterations in patients with chronic liver disease is extensively documented in cases of hepatic encephalopathy and minimal hepatic encephalopathy resulting from the development of cirrhosis and its subsequent evolution to hepatic insufficiency. However, with the onset of hepatitis C virus (HCV) infection, complaints of cognitive changes in patients without cirrhosis or significant hepatic impairment have been reported [1, 2].

Complaints of extrahepatic involvement in HCV infections are more frequent than those related to hepatic involvement since these occur late with the development of liver cirrhosis and its subsequent evolution to its decompensated form [3]. It is believed that 40 to 74% of patients infected with HCV will present with at least one extrahepatic manifestation in the course of their lives [4, 5]. Among the classifications of extrahepatic manifestations associated with C virus, neuropsychiatric complaints of fatigue, depression, and cognitive impairment are in category B of the scale proposed by Cacoub et al. [5] This category is defined by the high prevalence of these complaints present in this population, particularly when compared to the general population and associated with the absence of a defined pathophysiological mechanism that explains its occurrence.

Patients infected with HCV often report neuropsychiatric complaints even before the development of significant liver changes. Among the frequently described neuropsychiatric complaints are the presence of mild cognitive impairment, depression, and fatigue [6]. The prevalence of depression is estimated between 20% and 50% of patients infected with HCV, being higher than that found in the general population, which is approximately 10%. Another complaint that far exceeds the prevalence found in the general population is fatigue, with an estimated 50-80% of those infected with HCV. When considering studies published in the literature, it is observed that up to 50% of the patients presented a poor performance in neuropsychological tests, with the most significant effects observed in verbal episodic memory and working memory (a very short-term memory, which allow people to process information while engaged in other tasks, i.e., the type of memory that we used in mathematical calculations) [7].

The pattern of cognitive impairment described above involves the cortical regions of the frontal lobe and the nuclei of the base in a pattern similar to that observed in HIV infection [8]. However, despite evidence of the presence of cognitive dysfunction in chronic HCV patients, many studies have included patients with comorbidities associated with cognitive impairment, such as cirrhosis and depression, which serve as the confounding bias. The purpose of this text is to provide an overview of cognitive changes in patients with HCV.

## 2. Pathophysiology of Cognitive Impairment

The occurrence of central nervous system (CNS) involvement is seen in other viral infections, and human immunodeficiency virus (HIV) infection is one of the best studied to date. The family Flaviviridae to which HCV belongs has different types of viruses with recognized neurotropism, such as West Nile virus, Saint Louis encephalitis virus, Murray Valley virus, and Japanese encephalitis virus. Besides, yellow fever and dengue virus may occasionally result in encephalitis [9–11].

The fact that other members of the Flaviviridae family have recognized neurotropism, such as West Nile virus, is a frequent argument for explaining the nature of the hepatitis C virus in causing cognitive dysfunction. Since the discovery in 1920 of the viral cause of yellow fever, Flaviviridae family viruses have been documented as causes of arboviruses and liver disease in humans. Arboviral infections cause a broad spectrum of diseases, and after, acute infections can also progress to much more complex secondary conditions, resulting in long-term physical and cognitive impairment or early death [12].

The Flaviviridae family is currently classified into four genera: Flavivirus, Hepacivirus, Pestivirus, and Pegivirus. Although they belong to the same family and share structural features and strategies of replication in common, there is a great phylogenetic distance between members of the genus Hepacivirus to which the HCV belongs and arbovirus belonging to the genus Flavivirus [13, 14]. Therefore, although they belong to the same family, there are marked differences between the viruses of these two genera, which makes it impossible to establish a relation of similarity between the two regarding the involvement of the central nervous system.

Although the exact mechanism that would result in the cognitive changes observed in patients with hepatitis C is not known, some proposals have been made to explain this occurrence:

1. The “Trojan Horse” effect: this hypothesis considers the possibility of HCV entering the brain through the infection of circulating monocytes in the blood. Monocytes are precursors of the microglia in the CNS, thus allowing HCV to reach the brain parenchyma (the so-called Trojan Horse phenomenon). The presence of HCV-infected microglia could result in the release of inflammatory interleukins such as tumor necrosis factor-alpha, interleukin-1, interleukin-6, and the release of neurotoxins such as nitric oxide and viral proteins, which would result in changes in cerebral function and cognitive alterations. This hypothetical scenario, which is similar to that postulated for HIV type 1 (HIV-1) infection, is corroborated by studies that demonstrated a close relationship between HCV sequences found in the cerebrospinal fluid and viral sequences found in the lymphatic system and peripheral blood mononuclear cells [1, 2, 15, 16]
2. The direct action of the virus through viral replication in neurons: the presence of HCV in the CNS has been described in several studies that used PCR-based techniques to detect the presence of the viral genome in the cerebral parenchyma and cerebrospinal fluid. In addition, some authors have reported the presence of negative-strand HCV RNA in the CNS, which suggests that there is viral replication in the brain parenchyma [1, 17, 18]
3. Effect of the inflammatory process: chronic HCV infection produces cytolytic effects on hepatocytes, culminating in the activation of the immune system. Therefore, the systemic inflammation produced could contribute to the observed brain dysfunction. This hypothesis is based on a growing body of evidence that demonstrates that the activation of the immune system in peripheral tissues can affect CNS functioning, causing affective, cognitive, and behavioral disorders. The presence of peripheral proinflammatory cytokines, such as interleukin-1 and interleukin-6, are the most likely mediators of these effects, penetrating the blood-brain barrier through active transport mechanisms, promoting the activation of the vagus nerve, and stimulating the neurotransmitter system. Chronic HCV-infected patients produce proinflammatory cytokines such as interleukin-6, interleukin-4, and tumor necrosis factor-alpha, which may result in cognitive impairment [2]
4. Blood-brain barrier endothelial cell infection: *in vitro* studies have shown that cerebral microvascular endothelial cells are susceptible to HCV infection. These cells are an integral part of the blood-brain barrier, and at least two independent derivatives of the cerebral microvascular endothelial cell line (hcmec/D3 and hbmec) express receptors on its external plasmatic membrane that allow HCV invasion. In addition to these data, immunohistochemical staining of the brain tissue revealed the presence of four receptors necessary for the entry of HCV into microvascular cells. SR-BI receptor expression is restricted to cerebral microvascular endothelium, suggesting that brain tropism occurs only through these cells [19].

Cellular apoptosis and reduction of the endothelial barrier activity were observed in cultures of endothelial cells infected with HCV. Thus, a possible mechanism proposed for the neuropathogenesis of HCV infection would be the direct viral invasion of endothelial cells, resulting in the apoptosis and breakdown of the blood-brain barrier that could allow inflammatory cytokines, viral particles, and other neurotoxic substances to enter the CNS, which would result in brain dysfunction and the neuropsychiatric changes reported by these patients. Astrocytes are in close contact with the basal lamina of endothelial cells, which secrete factors that help regulate their homeostasis. Probably, HCV endothelial cerebral infection may, therefore, impact astrocytic homeostasis, or even that HCV may also infect astrocytes by contiguity [1, 17, 18].

However, the presence of HCV RNA in brain tissue samples has to be considered with reservations, since it may be the result of contamination after the occurrence of the breakdown of the blood-brain barrier [20]. In addition, there is no correlation between viral load and the occurrence of cognitive deficits [2].

In the study conducted by Fishman et al., HCV RNA levels were quantified in multiple brain and liver samples from patients infected with the virus. It demonstrated that HCV levels in the brain tissue were 1,000 to 10,000 times lower than those found in the hepatic tissue [21]. This data suggests that the brain tissue is a less effective site for viral replication [20].

The most attractive hypothesis seems to be the infection of endothelial cells of the blood-brain barrier. Once infected, these cells would enter into apoptosis, resulting in the rupture of the blood-brain barrier, with the consequent entry of viruses, immune cells, and cytokines, resulting in neuroinflammation [1, 17–20].

## 3. Evidence of Cognitive Dysfunction in Patients with HCV

Approximately 50% of patients with hepatitis C have complaints of fatigue and cognitive deficits even before the development of significant hepatic impairment [2, 7, 21].

The first evidence of the occurrence of brain changes was discovered by Forton et al. who analyzed the cerebral metabolic relationships through the use of magnetic resonance spectroscopy in three distinct groups: (1) patients infected with HCV without the presence of significant fibrosis or cirrhosis (*n* = 30), (2) HBV-infected patients (*n* = 29), and (3) a group of healthy controls (*n* = 12). The authors found a statistically significant increase in the choline/creatine ratio in the white matter and basal ganglia of patients with HCV when compared to the other groups [22].

After this first publication, many other groups evaluated the impact of HCV infection on the development of cognitive impairment, as shown in Table 1.

Assessing the influence of HCV infection on the development of cognitive changes involves many challenges. The frequent presence of confounding factors such as illicit drug use, depression, and cirrhosis has the potential to produce cognitive impairment and therefore obscuring the role of HCV infection as a major actor in the development of cognitive impairment.

The presence of brain dysfunction in patients with liver cirrhosis is well known. Zeegen et al. described in 1970, through the use of neuropsychological tests, the occurrence of cognitive changes involving mainly psychomotor speed, attention, and executive function in cirrhotics who did not have clinical criteria for hepatic encephalopathy [23]. This condition is currently called minimal hepatic encephalopathy.

It is interesting to note that several studies have included in their samples individuals with liver cirrhosis or advanced stage of hepatic fibrosis. Also, other studies have not described the characteristics of their samples or used insensitive methods to exclude cirrhotic patients, as shown in Table 1.

The prevalence of depression in HCV patients is estimated to be up to 5 times more frequent than in the general population, being a critical point in carrying out studies that evaluate HCV infection and the occurrence of cognitive changes. The inclusion of people with major depression may be responsible for the low performance observed in several cohorts. However, the exclusion of depressed patients may result in the production of a representative sample of subjects who have few extrahepatic manifestations of HCV infection, which is a sensitive point and produces a significant dilemma in the design of studies that assess the cognitive manifestations of HCV infection. Despite these limitations, most studies point out to the existence of cognitive impairment in patients infected with HCV, such as the work carried out by Forton et al. and MacAndrews et al. [22, 24] In these two seminal studies, the post hoc multivariate analysis evidenced that the cognitive impairment was independent of the degree of depression.

In an attempt to eliminate confounding factors in the cognitive assessment of patients with HCV, the authors of the present review have used a rigorous selection criterion and applied an extended cognitive battery in a previous study. Exclusion of depression and advanced stages of liver fibrosis (F3 and F4 of the METAVIR classification) resulted in a group of HCV carriers with cognitive performance similar to age and educational level-paired control group [25]. However, the use of a low cut-off point in the Beck Depression Inventory (BDI) in this study, excluding cases whose symptoms only suggested mild forms of depression, may have resulted in selection bias and removed from the sample a representative population of patients with HCV. This previously published work found no evidence of an association between HCV infection and the presence of cognitive dysfunction, which corroborates the findings of few other studies [26–28].

The data presented in the literature indicate varying degrees of control over confounding factors, such as liver cirrhosis and depression. Such factors, combined with the use of different types of neuropsychological tests and the assessment of different domains of cognitive functions, provide great difficulty in comparing the results of preexisting studies.

As noted in Table 1, the literature available so far is composed of cross-sectional studies, which would not be an adequate design to establish a relationship of exposure by HCV as a causative factor of cognitive deficit. Cross-sectional studies is aimed only at determining the incidence or prevalence of a given condition. In addition, most studies recruited patients from tertiary hospitals, which poses the risk of selection bias, affecting the true prevalence of cognitive manifestations of HCV infection.

Despite all the exposed limitations, most studies have demonstrated the presence of cognitive changes, involving several functions such as attention and working memory, in a pattern that suggests the involvement of the frontal cortex and the basal ganglia, or the connections between these structures.

## 4. Conclusion

The literature consistently demonstrates the presence of cognitive deficits in patients with chronic HCV infection. However, the heterogeneous nature of published works with different degrees of control of confounding factors and designed as cross-sectional studies makes it impossible to categorically establish HCV infection as the sole responsible factor for the reported cognitive changes.

A large body of studies have established the presence of HCV penetration through the blood-brain barrier, HCV replication within the brain, and changes in neurocognitive tests when compared to controls, including works that demonstrated cognitive impairment independent of the degree of depression. However, the inclusion of confounding factors in several studies, the lack of standardization in the neuropsychological tests, and the absence of cohort studies preclude the assertion that HCV can cause brain dysfunction *per se.*

Future longitudinal studies with the use of neuropsychological and neurophysiological cognitive assessments before and after the use of direct-acting antivirals may provide further information on the role of HCV infection as a cause of cognitive impairment.

## Figures and Tables

**Table 1:** Cognitive assessment of HCV-infected patients.

Study	Study design	Sample	Measures	Main findings	Critique
Forton et al. [22]. (2002)	Cross-sectional	HCV: 27 HCV/PCR-: 16	D.C.R/T.T/C.T	Worst performance in the HCV group	Inclusion of depressed patients

Hilsabeck et al. [26] (2002)	Cross-sectional	HCV: 66 Hepatopathy of another cause: 14	R.C.F/D.C.T/T.T/C.T	No difference between the groups	Inclusion of patients with liver cirrhosis

Kramer et al. [29]. (2002)	Cross-sectional	HCV: 100 Group control: 100	P300 Minimental	Increased latency and reduced P300 amplitude in the group HCV	Inclusion of patients with liver cirrhosis

Córdoba et al. [28] (2004)	Cross-sectional	HCV: 40 HCV cirrhosis: 40 HCV D.C: 40 Group control: 40	A.V.L.T/T.T/C.T S.T/P.G/H.T	No difference between the groups	Inclusion of patients with liver cirrhosis

Weissenborn et al. [30] (2004)	Cross-sectional	HCV M.F: 15 HCV Moderate F: 15 Group control: 15	R.C.F/figure L.W.L.T/C.T	Worst cognitive performance of patients with HCV in cognitive fields involving attention and executive function	Inclusion of depressed patients

McAndrews et al. [24] (2005)	Cross-sectional	HCV: 37 Group control: 46	T.D/C.T/T.T S.T/S.T.W	Worst performance in the HCV group	Advanced hepatic fibrosis

Weissenborn et al. [31] (2006)	Cross-sectional	HCV: 16 HCV/PCR-: 4	Test d2 Battery tap	Cognitive change in 2/3 of individuals involving attention/concentration in the HCV group	High score in BDI

Karaivazglou et al. [27]. (2007)	Cross-sectional	HCV: 32 HBV: 29 Group control: 20	RAVLT/S.F.T T.T/C.T	No difference between the groups	Inclusion of patients with liver cirrhosis

Forton et al. [32]. (2008)	Cross-sectional	HCV: 16 HCV/PCR-: 18 Group control: 85-420	D.C.R	Change in the HCV + PCR + group involving working memory and attention when compared to the control and when compared to the HCV + PCR group	History of use of illicit drugs

Huckans et al. [33]. (2009)	Cross-sectional	HCV: 39 H.U.A.ID HCV: 24 N.H.A.ID Group control: 56	T.F.V/T.R.C.F/C.T/T.T	Cognitive change: attention, verbal memory, and psychomotor speed in the HCV group independent from the use of substances	Inclusion of depressed patients

Quarantini et al. [34]. (2009)	Cross-sectional	HCV: 33 HBV: 22	T.R.C.F/RAVLT; T.D; T.T	Worst performance of the HCV group in immediate memory in relation to the HBV group	Potential inclusion of patients with liver cirrhosis

Lowry et al. [35]. (2010)	Cross-sectional	HCV: 11 HCV PCR-: 9 Group control: 9	WMS III	The HCV group presented worst scores of memory and attention when compared to the control group	Inclusion of depressed patients

Farag et al. [36]. (2011)	Cross-sectional	HCV: 11 Group control: 14	W.C.T	The HCV group presented the worst results in the executive function tests and increase in the soluble receiver ii of the necrosis factor	Inclusion of patients with liver cirrhosis

Abrantes et al. [25] (2013)	Cross-sectional	HCV: 29 Group control: 29	Minimental T D.S/S.T/T.T B.N.T/T.F.V; T.D/G.F.D.T G.F.D.T/S.S.T/RAVLT	No difference between the groups	Strict exclusion criterion

Ibrahim et al. [37]. (2016)	Cross-sectional	HCV: 38 Group control: 210	Penn cognitive battery—Arabic version	Cognitive change involving various cognitive areas in the HCV group	Potential inclusion of patients with liver cirrhosis

Hilsabeck et al. [38] (2003)	Cross-sectional	HCV: 21	B.V.M.T-R/S.S.T/T.T/C.T	Cognitive changes: visuoconstructive, attention/concentration, visual scanning, psychomotor speed, and working memory	Inclusion of HIV patients, use of interferon and psychotropic drugs

Fontana et al. [39]. (2005)	Cross-sectional	HCV: 201	T.P-D/T.D T.T	33% presented dysfunction in the verbal memory and working memory	Inclusion of patients with liver cirrhosis

D.C.R: cognitive drug research; R.C.F: Rey Complex Figure; D.C.T: Digit Cancellation Test; T.T: Trail Testing; C.T: Code Testing; B.V.M.T-R: Brief Visuospatial Memory Test-Revised; S.S.T: Symbol Search Test; A.V.L.T: Auditory-Verbal Learning Test; S.T: Stroop test; P.G: Pegboard Grooved; H.T: Hooper Test; L.W.L.T: Luria Word List Test; I.N.F: interferon; D.C: decompensated cirrhosis; T.D: Digit Test; D-P.T: D-Prime Test; M.F: mild fatigue; Moderate F: moderate fatigue; W.C.T: Winconsin Card Test; RAVLT: Rey Auditory Verbal Learning Test; S.F.T: Semantic Fluency Test; V.F.T: Verbal Fluency Test; S.D.T: Simple Design Test; B.N.T: Boston Nomination Test/G.F.D.T: Geometric Figure Drawing Test; H.U.A.ID: history of use of alcohol and illicit drugs; N.H.A.ID: no history of alcohol and illicit drug use; BDI: Beck Depression Inventory.
